# Feeling Anxious Amid the COVID-19 Pandemic: Factors Associated With Anxiety Symptoms Among Nurses in Hong Kong

**DOI:** 10.3389/fpsyg.2021.748575

**Published:** 2021-10-01

**Authors:** Nelson Chun-yiu Yeung, Eliza Lai-yi Wong, Annie Wai-ling Cheung, Eng-kiong Yeoh, Samuel Yeung-shan Wong

**Affiliations:** ^1^The Jockey Club School of Public Health and Primary Care, Faculty of Medicine, The Chinese University of Hong Kong, Hong Kong, Hong Kong, SAR China; ^2^Centre for Health Systems and Policy Research, The Jockey Club School of Public Health and Primary Care, Faculty of Medicine, The Chinese University of Hong Kong, Hong Kong, Hong Kong, SAR China

**Keywords:** anxiety symptoms, COVID-19, nurses, work satisfaction, infection worry

## Abstract

**Background:** The coronavirus (COVID-19) pandemic has increased the burden for the medical systems around the world. In Hong Kong, the pandemic not only affects the local populations, but also the healthcare workers. Healthcare workers, especially nurses, involving in COVID-19 treatments are highly susceptible to adverse psychological outcomes (e.g., anxiety symptoms). Studies have shown that socio-demographic characteristics, COVID-19-specific worries, and work settings-related variables are associated with healthcare workers' well-being during the COVID-19 pandemic. However, relevant studies for nurses in Hong Kong are limited. This study examined the psychosocial correlates of anxiety symptoms among nurses in Hong Kong.

**Methods:** Nurses (*N* = 1,510) working in hospitals and community settings were recruited through nursing associations in Hong Kong between August 8, 2020 and September 22, 2020. They were invited to complete a cross-sectional survey measuring their anxiety symptoms, sociodemographic characteristics, COVID-19-specific worries, and satisfaction with work and workplace pandemic-control guidelines.

**Results:** 17.2% of nurses reported moderate to severe levels of anxiety symptoms. Results from hierarchical regressions found that higher COVID-19-specific worries (contracting COVID-19, family members contracting COVID-19 due to their nursing work, insufficient protective equipment at workplace) (β*s* ranged from 0.07 to 0.20, *ps* < 0.01), higher perceived stigma of being a healthcare worker (β = 0.18, *p* < 0.001), and lower work satisfaction (β = −0.21, *p* < 0.001) were associated with higher anxiety symptoms.

**Conclusion:** A moderate proportion of nurses in Hong Kong did report levels of anxiety symptoms amid the COVID-19 pandemic. Futures studies could focus on the contributing factors of anxiety symptoms to design for effective strategies to promote nurses' well-being during pandemic situations.

## Introduction

The Coronavirus pandemic (COVID-19) has become an international public health emergency, posing continuous threats to lives and healthcare systems worldwide. Since the first reported case on January 23, 2020, Hong Kong has reported 12,063 COVID-19 cases and 212 deaths as of August 23, 2021 (The Government of the Hong Kong Special Administrative Region, 2021). Studies have also found that the COVID-19 and its relevant control measures bring enormous psychological impacts (e.g., depression and anxiety) on the general population in Hong Kong (Choi et al., 2020). The COVID-19 pandemic is therefore highly stressful among people in Hong Kong.

Amid the COVID-19 pandemic, the needs of healthcare workers are largely neglected. Indeed, nurses have been identified as a segment of the healthcare worker population at higher physical and psychological risks due to exposure to patients' illness experience (Walton et al., 2020). Nurses' roles generally involve much closer contact with patients and spending longer time to serve patients' needs (Hamama-Raz and Minerbi, 2019). In Hong Kong, nurses have faced severe demands during the COVID-19 infection peaks (e.g., increased case load), plus experienced health-related worries about contracting COVID-19 and infecting their family members during the pandemic (Cheung et al., 2020). It is not surprising that local nurses are subject to high levels of anxiety symptoms. Understanding the potential determinants of nurses' well-being would help tailoring effective psychosocial interventions. This study aimed to examine the psychosocial correlates of Hong Kong nurses' anxiety symptoms during the COVID-19 pandemic.

### Potential Factors Associated With Anxiety Symptoms Among Nurses Amid the COVID-19 Pandemic

COVID-19-related stressors are likely to contribute to nurses' psychological well-being. For example, a prior study found that perceived risk of contracting COVID-19 was associated with anxiety symptoms, frontline nurses in China during COVID-19 pandemic (Cui et al., 2021). Working at hospitals and clinics during COVID-19 pandemic might increase nurses' worries about passing infection to family members being due to their jobs, which in turn affect healthcare workers' well-being (Li et al., 2020; Walton et al., 2020). It has also been found that healthcare workers' worries about insufficient personal protective equipment were associated with higher distress among healthcare workers in the United States (Hennein et al., 2021) and Korea (Han et al., 2021). Relevant studies are limited in the Hong Kong context. Therefore, we speculated that COVID-19-related worries would also be associated with more anxiety symptoms among local nurses in Hong Kong.

When facing highly stressful events like the COVID-19 pandemic, having different aspects of coping resources available could contribute to nurses' well-being (Labrague and De Los Santos, 2020). A recent review has supported that personal coping resources and work-related resources/risk factors are important determinants of psychological well-being among employees during the COVID-19 crisis (Rigotti et al., 2021), including healthcare workers. In this study, we aimed to examine the roles of interpersonal and work-related coping resources in anxiety symptoms among nurses in Hong Kong.

Regarding interpersonal resources, emerging research has started to examine the role of perceived stigma in healthcare workers' well-being during the COVID-19 pandemic. Due to the job nature, it is inevitable for healthcare workers to have the close contact with suspected and diagnosed cases of COVID-19 in the pandemic situation. Healthcare workers are commonly regarded as individuals with high risks for infecting and transmitting the virus. In the contexts of prior pandemic situations like the Severe Acute Respiratory Syndrome (SARS) and the Middle East Respiratory Syndrome (MERS), perceived stigma has been found to be a significant contributor of poor psychological well-being among healthcare workers (Gupta and Sahoo, 2020). Specific to the context of COVID-19, perceived stigma was associated with adverse mental health outcomes (e.g., psychological distress, probable depression) among healthcare workers in Bangladesh (Khan et al., 2021) and the US (Hennein et al., 2021). Health professionals' exposures to stigma or discriminatory behaviors may lead to gradual physical and psychological deterioration (Ramaci et al., 2020). In Hong Kong, healthcare workers are generally highly respected professions (Schoeb, 2016). It is important to examine how perceived stigma of being a healthcare worker contribute to local nurses' well-being in the context of COVID-19.

Research also suggests that work-related coping resources (e.g., level of job satisfaction, pandemic control measures at the institutions) can be associated with well-being among healthcare workers. Studies have found that individuals who are satisfied with work are more likely to find meaning in nature of work, which facilitate well-being during the COVID-19 pandemic (Hamama-Raz et al., 2021). Another study found that higher work satisfaction was associated with perceptions of positive changes (e.g., secondary posttraumatic growth) from the work experience during the pandemic among paramedics and nurses in Poland (Ogińska-Bulik et al., 2021a). The protective role of work satisfaction in anxiety symptoms among nurses should be further examined. On the other hand, workplace arrangements matter in employees' well-being during the pandemic. For example, implementation of COVID-19-related accommodating measures (e.g., introduction of reliable information resources, preventive measures to reduce risk of infection at workplace) were associated with lower psychological distress and better job performance among a heterogeneous sample of Japanese employees from multiple industries (Sasaki et al., 2020). Having transparent and timely policies for prevention of nosocomial infections (e.g., healthcare-associated infections) at the hospitals/clinics was associated with lower risks for probable depression and anxiety among healthcare workers in the US (Hennein et al., 2021). Whether satisfaction with workplace pandemic control guidelines could contribute to anxiety symptoms among local nurses has yet to be explored. Based on the above-mentioned literature, we expected that work satisfaction and satisfaction with workplace pandemic control guidelines would be associated with lower anxiety symptoms among Hong Kong nurses during the COVID-19 pandemic.

### Purpose and Hypotheses

This study examined the psychosocial correlates of anxiety symptoms among nurses in Hong Kong amid the COVID-19 pandemic. Based on the aforementioned studies, we hypothesized that higher levels of COVID-19 worries and perceived stigma, plus lower levels of work-related resources (work satisfaction, satisfaction with workplace pandemic control guidelines) were associated with higher anxiety symptoms.

## Methods

### Participants and Recruitment Strategies

The nurses working in either public or private service provision in different settings (including inpatient, outpatient, outreach service in community setting) were eligible for this study. Those who were nursing trainees and retired nurses were excluded from the study sample. All registered members of the Association of Hong Kong Nursing Staff (*n* = 16,500), the labor union of nurses in Hong Kong, were approached and invited to this study using their email contacts. The self-administered questionnaire was distributed to the nurses in an internet-based link along with an invitation email. An information sheet about the study was included at the beginning of the questionnaire, followed by an electronic consent form. The participants who agreed to join the study filled in the questionnaire on their own electronic devices. A reminder for participation into the survey was sent 2 weeks after the first invitation email. Responses from 1,566 participants were collected *via* the online platform. Among them, 56 did not fulfill the eligibility criteria (e.g., not being a nurse, being retired, or being a nursing student). Therefore, only 1,510 valid responses representing working nurses included both worked as full-time or part-time in health care settings were retained in the analyses.

Comparison of sample characteristics was made with statistics of nurse population in Hong Kong to explore the potential selection bias. Although the response rate was low (1,510/16,500), characteristics of this sample were matched to nurse population in Hong Kong according to the latest statistics from Department of Health (Department of Health, 2016).

Upon completing the questionnaire, participants received HKD$50 (approximately USD$6.43 for compensation of their time. The study was conducted between August 8, 2020—September 22, 2020 (when there were 5,059 patients diagnosed and 105 dead from COVID-19 in Hong Kong). The study protocol was approved by the Research Ethics Committee (CUHK-NTEC CREC) at the first author's institution (Protocol no. CRE-2020.073).

### Measures

#### Anxiety Symptoms

The General Anxiety Disorder-7 (GAD-7) was used to measure participant's levels of anxiety over the last 2 weeks (Spitzer et al., 2006). On a 4-point Likert scale (0 as *not at all* to 3 as *nearly every day*), a higher sum score from all items (e.g., “feeling nervous, anxious, or on edge”) indicated more frequent anxiety symptoms. The Chinese version of the GAD-7 has been shown to be reliable and valid in community populations in Hong Kong, with a cut-off point of ≥10 indicating at least a moderate level of anxiety (Choi et al., 2020). The Cronbach's alpha for this sample was 0.91.

#### COVID-19 Specific Worries

Three items were specifically developed to measure participants' worries about the consequences of getting COVID-19 (i.e., “I worry that I would infect with COVID-19 from work,” “I worry that my family members would infect with COVID-19 because of my work,” and “I worry that the protective equipment at my workplace is not sufficient”). On a 5-point scale (1 as *not at all true*, 5 as *always true*), higher item scores represented higher COVID-19-related worries.

#### Perceived Stigma

Three items were specifically developed to measure participants' perceived stigma of being a healthcare worker during the COVID-19 pandemic (i.e., Because of my job, I felt being stigmatized by (1) my family/relatives, (2) my neighbors, and (3) friends at social gatherings. On a dichotomous scale (1 as *yes*, 0 as *no*), a higher sum score represented higher perceived stigma of being a healthcare worker. A similar scoring method was also used in another study measuring perceived stigma among healthcare workers in Columbia (Campo-Arias et al., 2021). We conducted a supplementary exploratory factor analysis to examine the dimensionality of the items. One factor was extracted through principal component analysis with orthogonal rotation, explaining 42.1% of variances in perceived stigma. Those results supported that this concept was suitable to be represented in a single dimension for the subsequent regression analysis.

#### Work Satisfaction

One item was used to measure participants' work satisfaction during the COVID-19 pandemic (“how much do you feel satisfied with my current job?”), on a 5-point scale (1 as *strongly unsatisfied*, 5 as *strongly satisfied*). A higher score indicated a higher level of job satisfaction. Prior research has supported the validity of this single item measure in predicting health outcomes among employees in different countries (Dolbier et al., 2005).

#### Satisfaction With Workplace Pandemic Control Guidelines

Five items were developed to measure participants' satisfaction with pandemic control guidelines at their workplace. Participants were asked to rate their level of satisfaction toward the different aspects of the workplace pandemic control guidelines (i.e., comprehensiveness, clarity, timeliness, transparency, and efficacy), on a 5-point scale (1 as *strongly unsatisfied*, 5 as *strongly satisfied*). A higher mean score from the item responses represented a higher level of satisfaction of workplace pandemic control guidelines. We also conducted a supplementary exploratory factor analysis to examine the dimensionality of the items. One factor was extracted through principal component analysis with orthogonal rotation, explaining 79.8% of variances in satisfaction with workplace pandemic control guidelines. Those results supported that this concept was suitable to be represented in a single dimension for the subsequent regression analysis. The Cronbach's alpha of the scale in this sample was 0.94.

#### Sociodemographic and Job-Context Variables

Socio-demographic variables (e.g., age, years in the profession, marital status, religious affiliation) and job-context variables (e.g., working in a team specifically caring for COVID-19 patients and suspected cases (aka “dirty team”) were measured.

### Analytic Plan

Descriptive statistics and Pearson correlations among the major variables were computed. Internal consistencies of the scales were indicated by their corresponding Cronbach's alphas. Hierarchical regressions were conducted to examine the associations between the independent variables and anxiety symptoms. The sequence of entering independent variables followed the suggestions from prior studies on individuals' well-being in response to emerging infectious diseases and other traumatic events (Yeung et al., 2016, 2021). In the first block, background variables showing significant associations with anxiety symptoms in bivariate correlations were entered. In the second and third blocks, COVID-19 worries, and perceived stigma were entered in the model, respectively. In the last block, work-related coping resources (i.e., work satisfaction, satisfaction with workplace pandemic control guidelines) were entered. The analyses were conducted using SPSS 26.0.

### Sample Size Planning

Expecting a small-to-medium effect size (*f* ^2^ = 0.05) in the association between the independent variables and anxiety symptoms in the hierarchical regression analysis, we needed at least 515 participants to achieve a statistical power of 0.95 at *p* = 0.05 (G^*^Power 3.1.2). With our sample size (*N* = 1,510), we should be able to detect the expected effect size with sufficient statistical power.

## Results

### Participants' Characteristics

Most of the participants were aged between 30 and 39 (36.8%) and married (50.7%). On average, they have been working in the profession for around 8.9 years (SD = 8.60). About one-fifth of the participants were specifically taking care for diagnosed and suspected cases of patients with COVID-19 (‘dirty team”) (18.5%) and reporting at least a moderate level of anxiety (GAD ≥ 10) (17.2%) (Table 1).

**Table 1:** Characteristics of the participants (*N* = 1,510).

	**Frequency (%)/Mean (SD)**
Age group	
18–25	381 (25.2%)
26–35	555 (36.8%)
36–45	354 (23.4%)
46–55	210 (13.9%)
Above 55	10 (0.7%)
Marital status	
Single	692 (45.8%)
Married	765 (50.7%)
Separated/Divorced/Widowed	53 (3.5%)
Having a religious affiliation	552 (36.6%)
Years in the profession	8.91 (8.560)
<1 year	82 (5.4%)
1–3 years	315 (20.9%)
3–5 years	226 (15.0%)
5–10 years	383 (25.3%)
10–20 years	248 (16.4%)
More than 20 years	256 (17.0%)
Working in the team caring for COVID-19 patients and	280 (18.5%)
suspected cases (i.e., “dirty team”)	
Probable anxiety (GAD-7 ≥10)	256 (17.2%)

### Correlations Between Independent Variables and Anxiety Symptoms

The correlation analysis results showed that higher levels of COVID-19-specific worries (infecting with COVID-19, family members infecting with COVID-19 due to the participants' nursing duties, insufficiency of protective equipment at workplace), higher perceived stigma, lower work satisfaction, and lower satisfaction with workplace pandemic control guidelines were correlated with higher anxiety symptoms (*r*s ranged from 0.12 to 0.39, *p* < 0.05) (Table 2). Regarding socio-demographic variables, younger age, fewer years in the profession, not being married, having children (*r*s ranged from −0.15 to −0.09, *ps* < 0.001), and working in the “dirty team” were correlated with more anxiety symptoms (*r* = 0.12, *p* < 0.001). Those variables were included as covariates in the subsequent regression analysis. Other socio-demographic variables reported non-significant correlations with anxiety symptoms (*ps* > 0.05, not tabulated).

**Table 2:** Descriptive statistics and correlations among major variables (*N* = 1,510).

	**1**	**2**	**3**	**4**	**5**	**6**	**7**	**8**	**9**	**10**	**11**	**12**
1. Anxiety symptoms (GAD-7)	–											
2. Age^†^	−0.15^***^	–										
3. Years in the profession	−0.10^***^	0.65^***^	–									
4. Marital status^†^	−0.09^***^	0.41^***^	0.37^***^	–								
5. Having children^†^	−0.09^***^	0.43^***^	0.31^***^	0.66^***^	–							
6. Dirty team^†^	0.12^***^	−0.10^***^	−0.03	−0.10^***^	−0.11^***^	–						
7. Worry about infecting with COVID-19	0.45^***^	−0.29^***^	−0.23^***^	−0.11^***^	−0.11^***^	0.16^***^	–					
8. Worry about family members infecting with COVID-19	0.43^***^	−0.27^***^	−0.21^***^	−0.11^***^	−0.11^***^	0.14^***^	0.80^***^	–				
9. Worry about insufficient protective equipment at workplace	0.35^***^	−0.26^***^	−0.18^***^	−0.13^***^	−0.14^***^	0.12^***^	0.54^***^	0.52^***^	–			
10. Perceived stigma	0.32^***^	−0.10^***^	−0.09^***^	−0.07^**^	−0.10^***^	0.18^***^	0.25^***^	0.23^***^	0.21^***^	–		
11. Work satisfaction	−0.39^***^	0.23^***^	0.15^***^	0.15^***^	0.15^***^	−0.12^***^	−0.36^***^	−0.35^***^	−0.34^***^	−0.22^***^	–	
12. Satisfaction with workplace pandemic control guidelines	−0.32^***^	0.31^***^	0.20^***^	0.18^***^	0.22^***^	−0.09^***^	−0.40^***^	−0.39^***^	−0.48^***^	−0.19^***^	0.53^***^	–
Mean	5.79	2.28	8.91	0.51	0.38	0.19	3.15	3.48	3.08	0.40	3.33	3.14
SD	4.65	1.01	8.60	0.50	0.49	0.39	0.89	1.02	1.01	0.64	0.87	0.89

* *p ≤ 0.05*,

** *p ≤ 0.01, and*

*** *p ≤ 0.001*.

† *Age: 18–29 (1), 30–39 (2), 40–49 (3), 50–59 (4), 60–69 (5); Marital status: Married (1), Single/separated/widowed (0); Having children: Yes (1), No (0); Dirty team: Yes (1), No (0)*.

### Hierarchical Regression Analysis

Given that the independent variables were moderately correlated with each other, the independent variables were checked for multicollinearity in the regression analysis. We did not find any variables reporting a variance inflation factor (VIF) ≥ 4, supporting the absence of multicollinearity. In Block 1, the background variables explained 3.3% of variance in anxiety symptoms. Specifically, younger age (β = −0.12, *p* < 0.01) and working in the “dirty team” (β = 0.10, *p* < 0.01) were significantly associated with higher anxiety symptoms. In Block 2, higher levels of worries about contracting COVID-19 from work, about family members contracting COVID-19 due to their nursing duties, and about insufficient protective equipment at workplace (β*s* ranged from 0.13 to 0.25, *ps* < 0.001) were significantly associated with higher anxiety symptoms. In Block 3, higher perceived stigma of being a healthcare worker was associated with higher anxiety symptoms (β = 0.20, *p* < 0.001), explaining an additional 3.7% of variance. In Block 4, work-related coping resources variables explained an additional 3.8% of the variance in anxiety symptoms. Only work satisfaction was associated with lower anxiety symptoms (β = −0.21, *p* < 0.001). With all the independent variables, the overall model explained 30.4% of variances in anxiety symptoms (Table 3).

**Table 3:** Hierarchical regression analyses explaining anxiety symptoms (*N* = 1,510).

	**Anxiety Symptoms (GAD-7)**	
	**β**	**Δ*R*^**2**^**
Block 1—Sociodemographic and job-context variables		0.033^***^
Age^†^	−0.12^**^	
Years in the profession	−0.01	
Marital status^†^	−0.01	
Having children^†^	−0.02	
Dirty team^†^	0.10^***^	
Block 2—COVID-19 worries		0.196^***^
Age^†^	0.00	
Years in the profession	0.03	
Marital status^†^	−0.01	
Having children^†^	−0.03	
Dirty team^†^	0.03	
Worry about infecting with COVID-19	0.25^***^	
Worry about family members infecting with COVID-19	0.15^***^	
Worry about insufficient protection equipment	0.13^***^	
Block 3—Perceived stigma		0.037^***^
Age^†^	−0.01	
Years in the profession	0.03	
Marital status^†^	−0.01	
Having children^†^	−0.02	
Dirty team^†^	0.01	
Worry about infecting with COVID-19	0.23^***^	
Worry about family members infecting with COVID-19	0.14^***^	
Worry about insufficient protection equipment	0.12^***^	
perceived stigma of being a healthcare worker	0.20^***^	
Block 4—Work-related resources		0.038^***^
Age^†^	0.02	
Years in the profession	0.03	
Marital status^†^	−0.00	
Having children^†^	0.01	
Dirty team^†^	0.00	
Worry about infecting with COVID-19	0.20^***^	
Worry about family members infecting with COVID-19	0.12^***^	
Worry about insufficient protection equipment	0.07^**^	
perceived stigma of being a healthcare worker	0.18^***^	
Work satisfaction	−0.21^***^	
Satisfaction with workplace pandemic control guidelines	−0.03	
Total *R*^2^		0.304

* *p ≤ 0.05*,

** *p ≤ 0.01, and*

*** *p ≤ 0.001*.

† *Age: 18–29 (1), 30–39 (2), 40–49 (3), 50–59 (4), 60–69 (5); Marital status: Married (1), Single/separated/widowed (0); Having children: Yes (1), No (0); Dirty team: Yes (1), No (0)*.

## Discussion

This was one of the first studies in examining the psychosocial correlates of anxiety symptoms among nurses in Hong Kong amid the COVID-19 pandemic. We found that 17.2% of nurses in Hong Kong reported moderate-to-severe levels of anxiety symptoms (GAD-7 ≥10). Using the same measurement scale, our sample reported a comparable prevalence of probable anxiety (GAD-7 ≥10) with healthcare workers in India (Wilson et al., 2020), and China (Liu et al., 2021); but slightly lower than those in Iran (Pouralizadeh et al., 2020), the United Kingdom (Choudhury et al., 2020), and the United States (Kim et al., 2021). Regional differences in the severity of the pandemic and the time of conducting the study might contribute to the discrepancies.

Not surprisingly, we found that different aspects of COVID-19 worries (including worries of contracting COVID-19, family members being infected with COVID-19 from participants' nursing duties, insufficiency of personal protection equipment at workplace) were independently contributing to anxiety symptoms among the nurses in Hong Kong. Similar findings have been reported in healthcare workers in other countries, including China (Zhang et al., 2021), Korea (Han et al., 2021), and the US (Hennein et al., 2021). Providing nurses with safe and secure work environments and sufficient supply of personal protective equipment is required for protection of mental health among nurses coping with the pandemic. Moreover, it will still be important to examine other aspects of worries and their contributions to those workers' well-being. Research has found that healthcare workers in the US and UK expressed concerns about the access to support for personal and family needs (e.g., childcare, lodging, and transportation) with increasing working hours and demands, about the capability of providing competent medical care if deployed to a new unit, and dealing with emotional reactions of patients (Cipolotti et al., 2020; Shanafelt et al., 2020). Having more comprehensive measurements about COVID-19-related worries will further inform health organizations/institutions about what issues they should address to serve the needs of the healthcare workers.

Even of the level of perceived stigma was not high in the sample (mean = 0.40 out of 3) and healthcare workers are generally highly respected professions in Hong Kong (Schoeb, 2016), perceived stigma was associated with higher anxiety symptoms among those nurses. Given that the study was conducted in relatively early stage of the pandemic (August–September 2020) with higher reliance on social distancing measures and personal hygiene (when there was no vaccination campaign rolling out yet), public fear about contracting COVID-19 from healthcare workers was still prevalent. Such fear might drive stigmatizing behaviors among the public and relatives (e.g., avoidance toward their acquaintances and neighbors who worked as healthcare workers) (Stangl et al., 2019). The finding has been in line with studies on healthcare workers in Bangladesh (Khan et al., 2021), Korea (Han et al., 2021), and the US (Hennein et al., 2021), implying that such phenomenon might be culturally universal. In addition, it is noteworthy that nurses who worked in the “dirty team” were more likely to report a higher level of perceived stigma. Such observations were also consistent with studies in Bangladesh (Khan et al., 2021) and Egypt (Mostafa et al., 2020) showing that healthcare workers who directly cared for COVID-19 patients reported higher perceived stigma than their counterparts who did not. Given that exposure to stigma or discriminatory behaviors may lead to gradual physical and psychological deterioration among healthcare workers (Ramaci et al., 2020), greater psychosocial support should be provided for healthcare workers, especially those at a higher risk of stigmatization.

Consistent with some prior studies (Hamama-Raz et al., 2021; Hennein et al., 2021; Ogińska-Bulik et al., 2021b), nurses who experienced lower work satisfaction were more likely to report more anxiety symptoms. The benefits of work satisfaction might also be associated with people's coping strategies toward work-related stressors. For example, a study in Poland found that higher work satisfaction was associated with more adaptive coping strategies (e.g., positive reframing, acceptance) among healthcare workers providing care for trauma victims in the hospitals (Ogińska-Bulik et al., 2021a). Those strategies have also been found to be associated with better psychological well-being among healthcare workers in the COVID-19 pandemic situations (Mong and Noguchi, 2021).

Even satisfaction of workplace pandemic control guidelines was bivariately correlated with fewer anxiety symptoms, it did not emerge as a significant contributor after accounting for other variables. It implied that COVID-19 worries, and work satisfaction might be even more important factors associated with anxiety symptoms among the nurses. To better understand how working environment might affect healthcare workers' adjustments to the COVID-19, it will still be important to further examine other aspects of work settings and arrangements and their contributions to those workers' well-being. For example, a previous study showed that satisfaction with the workload and the capability of participating in decision making were associated with fewer anxiety symptoms, whereas satisfaction with monetary compensation was associated with lower perceived stress among public health doctors in Korea (Han et al., 2021). Having more detailed analysis on the roles of specific aspects of work arrangements will benefit health organizations/institutions to adjust their measures to serve the needs of the healthcare workers.

### Limitations

This study had several limitations. First, it was a cross-sectional study so that the tested relationships were not causal. Future studies could examine how the changes in the independent variables could predict anxiety symptoms temporally using longitudinal designs. Second, all of the measures were self-reported, which might be subject to recall bias. However, the use of self-reported measures is common among many studies conducted during the COVID-19 pandemic (Salari et al., 2020) due to its convenience, low cost, and ease of administration at pandemic situations. Third, we recruited nurses through nursing associations in Hong Kong, where self-selected bias might be apparent. Comparable to response rates in some prior studies targeting at healthcare workers conducted online during the COVID-19 pandemic (Alenazi et al., 2020; Ammar et al., 2020), our response rate was low at 9.2% (1,510/16,500). Despite the low response rate, characteristics of this sample were matched to nurse population in Hong Kong according to the latest statistics from Department of Health (Department of Health, 2016). Readers should be noted that the findings might not be fully generalizable to the nurses and healthcare professionals in other countries with different medical systems. Fourth, the model only explained a moderate proportion of variance in anxiety symptoms. Other factors may be at play. For example, personality attributes (e.g., resilience, Type D personality) (Labrague and De Los Santos, 2020; Tuman, 2021), coping strategies (Si et al., 2020; Özçevik Subaşi et al., 2021), and impact of the pandemic on family roles have been found to be important contributors to people's well-being during COVID-19 (Cipolotti et al., 2020; Luceño-Moreno et al., 2020). Considering these variables could allow a more comprehensive capture the contributors to people's mental health outcomes in response to the pandemic. Fifth, this study targeted on an underserved population in the literature. Some scales and items (e.g., COVID-19 distress, work-related stress) were newly developed for the context of COVID-19, which were yet to be fully validated in this population. Specifically-developed items were also commonly used as predictors of mental health outcomes among different Asian populations during the COVID-19 pandemic (Choi et al., 2020; Yeung et al., 2020). Even our self-developed scales reported satisfactory psychometric properties, validating our findings by measuring the same concepts (e.g., Work Satisfaction Scale, perceived stigma for healthcare workers) (Traynor and Wade, 1993; Mostafa et al., 2021) or similar concepts (e.g., COVID-19 organizational support) (Zhang et al., 2020) with other standardized scales is worthwhile.

### Implications

This study highlighted that COVID-19 worries, perceived stigma, and work satisfaction might be prominent contributors to anxiety symptoms among those nurses. The World Health Organization recommends that healthcare workers should maintain self-care, be attentive to their own mental health problems, and reflect on how their experiences may influence themselves and their loved ones. Identifying and instituting effective treatment strategies to improve psychological outcomes for nurses is essential. Practically, our findings also implied that alleviating COVID-19 worries, addressing stigma toward healthcare workers, and increasing work satisfaction might be important intervention strategies to reducing anxiety symptoms among the nurses amid the COVID-19 pandemic. Development of digital interventions targeting at healthcare workers' well-being has also been emerging. Recently in the United Kingdom, a free digital education package has been developed to provide valuable information to enhance healthcare workers' skills in coping with social stigma, self-care, and social support seeking during the pandemic (Blake et al., 2020). On the other hand, experimental studies also indicated that expressive writing interventions could be effective in improving psychological health among Italian healthcare workers during the COVID-19 pandemic (Procaccia et al., 2021). Tailoring an intervention for local healthcare workers based on our findings, it is worth exploring if similar mobile applications providing additional modules to allow individuals to reflect on positive work experience and meaning derived from job duties, plus express negative emotions related to stigmatization experience would facilitate well-being.

## Conclusion

A moderate proportion of nurses in Hong Kong did report at least moderate levels of anxiety symptoms amid the COVID-19 pandemic. This study highlighted that COVID-19 worries, perceived stigma, and work satisfaction were the important contributors to anxiety symptoms among those nurses. We hope our findings could help tailoring intervention strategies to promote nurses' well-being during pandemic situations.

## Data Availability Statement

The datasets presented in this article are not readily available because their containing information that could compromise the privacy of research participant. The datasets are available from the corresponding author on reasonable request. Requests to access the datasets should be directed to lywong@cuhk.edu.hk.

## Ethics Statement

The studies involving human participants were reviewed and approved by the Joint Chinese University of Hong Kong—New Territories East Cluster Clinical Research Ethics Committee. The patients/participants provided their written informed consent to participate in this study.

## Author Contributions

NY and EW conceptualized the study and led on the manuscript writing. AC and NY collected and analyzed the data. E-kY and SW provided feedback on drafts of the manuscript. All authors approved the final manuscript draft.

## Conflict of Interest

The authors declare that the research was conducted in the absence of any commercial or financial relationships that could be construed as a potential conflict of interest.

## Publisher's Note

All claims expressed in this article are solely those of the authors and do not necessarily represent those of their affiliated organizations, or those of the publisher, the editors and the reviewers. Any product that may be evaluated in this article, or claim that may be made by its manufacturer, is not guaranteed or endorsed by the publisher.
